# False-Positive Asymmetrical Tongue Muscle 18F-FDG Uptake in Hypoglossal Nerve Paralysis Following Lymph Node Dissection in a Pediatric Patient with Malignant Rhabdoid Tumor of the Neck

**DOI:** 10.3390/children11030348

**Published:** 2024-03-15

**Authors:** Yuta Matsumoto, Motohiro Matsui, Akari Makidono, Atsushi Makimoto, Yuki Yuza

**Affiliations:** 1 Department of General Pediatrics, Tokyo Metropolitan Children’s Medical Center, Tokyo 183-8561, Japan; 2 Department of Pediatric Hematology Oncology, Tokyo Metropolitan Children’s Medical Center, Tokyo 183-8561, Japanatsushi_makimoto@tmhp.jp (A.M.);; 3 Department of Radiology, Tokyo Metropolitan Children’s Medical Center, Tokyo 183-8561, Japan

**Keywords:** combined positron emission tomography–computed tomography, PET-CT, malignant rhabdoid tumor, false-positive 18F-fluorodeoxyglucose uptake

## Abstract

Background: Although positron emission tomography combined with computed tomography (PET-CT) plays an important role in detecting various types of childhood malignancy, it has low positive predictive value, owing to the nonspecific uptake of 18F-fluorodeoxyglucose (FDG) by normal tissue in various benign conditions. Case summary: A 5-year-old male patient with a malignant rhabdoid tumor originating in the left neck underwent primary tumor resection concurrently with ipsilateral lymph node dissection after receiving neoadjuvant chemotherapy consisting of cyclophosphamide, carboplatin, etoposide, vincristine, and doxorubicin. He later received the same adjuvant chemotherapy as well as proton therapy for the primary tumor. Sixteen months after completing the initial therapy, follow-up PET-CT revealed a novel area of glucose hypermetabolism in the right side of the tongue, which was suspected of being a recurrence. However, a physical examination and magnetic resonance imaging (MRI) demonstrated no evidence of tumor recurrence. The patient had a significant leftward deviation of the tongue, suggesting left hypoglossal nerve paralysis. Denervation of the ipsilateral intrinsic tongue muscles secondary to the treatment had caused atrophy in the ipsilateral muscles and compensatory hypertrophy in the contralateral muscles, which increased FDG uptake. Physicians should carefully confirm any diagnosis of a locally recurrent tumor because PET-CT often produces ambiguous findings.

## 1. Introduction

Positron emission tomography combined with computed tomography (PET-CT) plays an important role in the surveillance of the recurrence of various types of childhood malignancy. However, physicians should be cautious when interpreting PET-CT findings because 18F-fluorodeoxyglucose (FDG) can accumulate in both physiologically (in the salivary glands, urinary tract, heart, and skeletal muscles) and pathologically (in inflammatory and infectious conditions) benign tissues [1]. This peculiarity of PET-CT can produce false-positive findings, leading to the misdiagnosis of cancer recurrence.

One example of such a finding is the compensatory hypertrophy of the tongue, which results from nerve paralysis induced by a tumor or an invasive treatment for the tumor and is often associated with head and neck malignancies in adult patients [2,3]. We present herein a case of a pediatric patient who experienced left-sided, treatment-related denervation of the tongue muscles secondary to a multimodal treatment for a malignant rhabdoid tumor (MRT) in the left neck. This complication led to the atrophy of the ipsilateral muscles, limited tongue movement, and finally, compensatory hypertrophy of the contralateral muscles. The muscle asymmetry appeared on PET-CT as markedly increased FDG uptake in the right tongue, which was initially misdiagnosed as an MRT recurrence.

Extracranial MRT is a rare and highly aggressive malignant tumor that occurs in infants and toddlers. The annual incidence of extracranial MRT is 0.6 per million, with the highest incidence seen in infants less than 1 year old. Extracranial MRT occurs most commonly in the kidneys but can also occur in organs and soft tissues throughout the body. About 14% of cases are localized in the head and neck.

Due to the small number of cases, there is no established treatment for extracranial MRT, but multimodal treatment is often administered [4]. Its prognosis has been improving in recent years, but even with multimodal treatment, the three-year event-free survival rate and overall survival rate are 32.3% and 38.4%, respectively, indicating one of the poorest prognoses among pediatric malignancies [5]. Extra-cranial MRT recurrences are frequent and can occur either locally or at distant sites [6,7]. Unfortunately, their prognosis is even worse than that of the primary tumor [8].

Although recurrences of extracranial MRT are concerning, misinterpreting test results can lead to unnecessary, invasive testing, thereby increasing the physical and psychological burden on patients and their families. Hence, clinicians need to be aware of the possibility of false-positive and false-negative test results.

## 2. Case Presentation

A 5-year-old male patient with no remarkable medical history presented with a five-day history of a mass on the left side of the neck. He had no “B symptoms”, such as fever, drenching night sweats, or weight loss. A physical examination revealed a firm, painless mass approximately 5 cm in diameter in the left neck. Airway stenosis, restricted neck movement, and neurological symptoms, such as hoarseness, dysarthria, and dysphagia, were denied. The laboratory findings for the complete blood count, biochemistry, and coagulation and serum tumor markers, including neuron-specific enolase (NSE) and soluble interleukin-2 receptor (sIL-2R), were normal.

Ultrasonography initially revealed a mass with a heterogeneous, intra-tumoral echo in the left neck. Subsequently, magnetic resonance imaging (MRI) demonstrated an irregularly shaped mass with hyperintensity on T1-weighted imaging (T1WI), heterogeneous hyperintensity on T2-weighted imaging (T2WI), and restricted diffusion on diffusion-weighted imaging (DWI). The mass, measuring 54 × 48 × 33 mm, had compressed the left common carotid artery, left external carotid artery, left internal carotid artery, and left internal jugular vein. Enhanced CT demonstrated a regional lymph node metastasis. No distant metastatic lesion was detected.

An excisional biopsy specimen contained tumor cells with vesicular nuclei, prominent nucleoli, and eosinophilic cytoplasm. Based on these findings, a pretreatment, pathological diagnosis of malignant rhabdoid tumor (MRT) was made. Immunohistochemical analysis revealed diffuse, cytoplasmic-positive staining for vimentin and cytokeratin, positivity for CD56, CD99, and SMA, and negativity for BAF47 expression, confirming the previous diagnosis.

**Figure 1:**
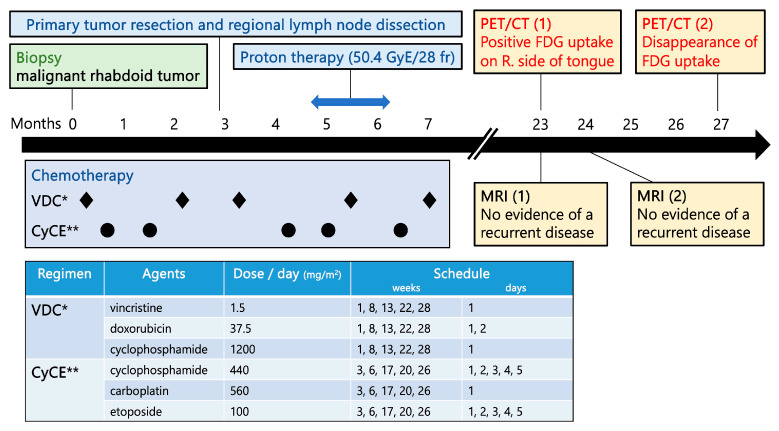
Timeline of the multimodal treatment and diagnostic procedures. Multimodal treatment, including chemotherapy as per the European Pediatric Soft Tissue Sarcoma Study Group (EPSSG) Non-Rhabdomyosarcoma Soft Tissue Sarcomas (NRSTS) 2005 [5], consisting of VDC* (vincristine, doxorubicin, and cyclophosphamide) and CyCE** (cyclophosphamide, carboplatin and etoposide); tumor resection with lymph node dissection; and proton beam therapy, was performed. Approximately 16 months later, PET-CT showed positive FDG uptake on the right side of the tongue, suggesting a recurrence. However, a series of MRI showed no evidence of a recurrence. The area first seen on PET-CT eventually resolved. p-Tx; duration after completion of chemotherapy.

After the biopsy, the patient received the following chemotherapy: cyclophosphamide, carboplatin, and etoposide (CyCE) on weeks 3, 6, 17, 20, and 26, and vincristine, doxorubicin, and cyclophosphamide (VDC) on weeks 1, 8, 13, 22, and 28 (Figure 1). The primary tumor was completely resected, and the patient underwent a modified radical neck lymph node dissection on all levels at week 12. Proton therapy (50.4 Gy) (RBE) was administered in 28 fractions to the primary tumor site and the regional lymph nodes in the left neck from weeks 19 to 25 concurrently with VC and CyCE.

**Figure 2:**
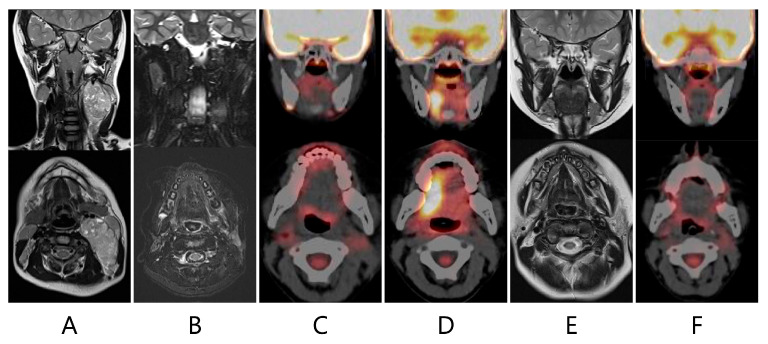
T2-weighted magnetic resonance imaging (MRI) showed a mass with heterogeneous signal intensity (54 × 48 × 33 mm) compressing the left carotid artery and internal jugular vein (**A**). MRI and 18F-FDG positron emission tomography combined with computed tomography (PET-CT) performed 9 (**B**) and 12 (**C**) months after completion of the chemotherapy found no recurrence. Sixteen months after completion of the chemotherapy, PET-CT revealed a novel area of glucose hypermetabolism in the right side of the tongue (SUV_max_ 10.79) (**D**). MRI performed at the same time demonstrated no evidence of a recurrence (**E**). Twenty months after completion of the chemotherapy, the FDG uptake in the tongue was no longer visible on PET-CT (**F**).

**Figure 3:**
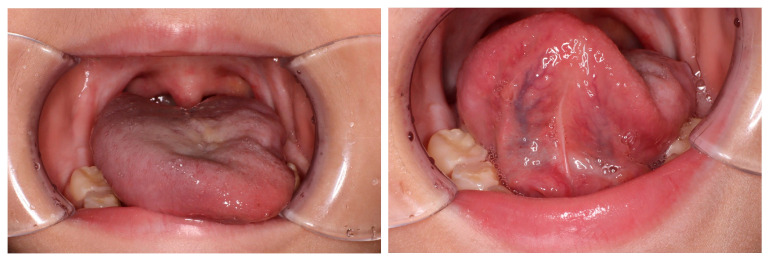
Physical examination at 16 months after completion of the initial therapy revealed atrophy of the left side of the tongue and deviation to the ipsilateral side indicating left hypoglossal nerve paralysis.

The patient underwent follow-up with neck MRI every five to six months and whole-body PET-CT every four to five months. Sixteen months after the completion of chemotherapy, PET-CT revealed a new area of glucose hypermetabolism in the right side of the tongue which appeared to be a recurrence (SUV_max_ 10.79) (Figure 2). However, at that time, MRI was unable to find any evidence of a recurrence, such as an irregular mass, which would be consistent with FDG uptake or a regional lymph node metastasis (Figure 2). A physical examination revealed atrophy of the left side of the tongue and ipsilateral deviation (Figure 3), suggesting left hypoglossal nerve paralysis. Taken together, the findings suggested that the FDG uptake in the right side of the tongue was not a recurrence but compensatory muscular hypertrophy secondary to the left hypoglossal nerve paralysis. Therefore, it was decided not to perform a biopsy but to continue monitoring the lesion with FDG accumulation with periodic PET-CT and MRI. When MRI was repeated one month later, no evidence of a recurrence was seen. Twenty months after the completion of the initial therapy, PET-CT demonstrated resolution of the FDG uptake in the right side of the tongue. However, the left hypoglossal nerve paralysis remained.

## 3. Discussion

Previous studies have reported several adult patients with symptoms similar to those of the present case. Timbang et al. reported a case of false-positive FDG uptake which occurred after treatment of head and neck cancer [3]. They concluded that radiotherapy for the tumor had caused ipsilateral hypoglossal nerve paralysis and that the false-positive FDG uptake had been caused by compensatory hypertrophy of the contralateral tongue muscle. Kamel et al. reported a case of false-positive FDG uptake in the laryngeal muscles on the side opposite to that of a Pancoast tumor, which induced laryngeal nerve paralysis on the same side as that of the tumor [9]. However, these reports provided little discussion of follow-up imaging findings, and no reports have, to date, described the resolution of FDG uptake resulting from compensatory processes after several months. The false-positive FDG uptake on PET/CT in pediatric patients can result from any one of a number of reasons, such as physiological uptake, infection, and inflammation [10]. As with PET-CT, other nuclear imaging modalities can also produce false-positive findings in pediatric patients [11]. A previous case report described false-positive uptake on a 123-I metaiodobenzylguanidine (MIBG) scan in a pediatric patient with pneumonia and neuroblastoma [12]. However, no previous study has reported a compensatory process as the cause. To the best of our knowledge, the present study is the first to report false-positive FDG uptake due to compensatory hypertrophy of lingual tissue in a pediatric cancer patient and to describe the relevant imaging findings.

As mentioned above, extracranial MRT has a poor prognosis due to its high malignancy potential, and the prognosis of recurrences is even worse. Furthermore, although there are no reports of a metastasis of extracranial MRT to the tongue, it can theoretically occur and result in restriction of tongue mobility [13]. Hence, it is paramount to detect a recurrence as early as possible or to determine carefully whether or not a lesion is a recurrence. In the present case, the FDG uptake in the area contralateral to the primary tumor after the initial therapy, which included chemotherapy, surgery, and radiotherapy, made it difficult to distinguish between a compensatory process and a recurrence.

The neck is a complex area with numerous vessels, muscles, and nerve fibers, as mentioned above [14]. Childhood head and neck cancers are rare but have recently been on the rise [15,16]. They are often detected by a swelling in the neck [17], which may be accompanied by neurological symptoms, such as hoarseness, dysarthria, or dysphagia due to nerve compression by the tumor [18]. Furthermore, since invasive treatments can give rise to these neurological symptoms, it is important to determine whether the symptoms are caused by the tumor itself or by the treatment.

In the present case, the invasive primary tumor in the left neck, which necessitated surgical resection with regional lymph node dissection, was thought to be the main cause of the left hypoglossal nerve paralysis. The nerve paralysis in turn led to muscle atrophy through the increased workload of the contralateral side, thus restricting the tongue’s ipsilateral movements. Moreover, the muscles of the right side of the tongue enlarged through a compensatory process that led to increased local glucose consumption, which appeared as increased FDG uptake on PET-CT. The resolution of the FDG uptake after several months supports this finding. It is possible that the FDG uptake temporarily increased during the process of muscle cell proliferation caused by the resumption of the normal use of the tongue in speaking and eating.

The combination of multiple imaging modalities may help to prevent a misdiagnosis. In their meta-analysis assessing the performance of PET-CT in post-treatment response surveillance imaging of head and neck cancers in adult patients [19], Gupta et al. reported that PET-CT had high negative predictive value (NPV) (95.1%) but low positive predictive value (PPV) (58.6%). On the other hand, MRI enables high resolution imaging of soft tissue. Another study found that MRI, including diffusion-weighted imaging, had higher NPV and PPV (75% and 77%, respectively) than PET-CT (64% and 64%, respectively) in a surveillance of muscle invasion of head and neck cancers in adult patients [20]. In the surveillance imaging of cancers in children and young adults, whole-body, ferumoxytol-enhanced, diffusion-weighted MRI may be more useful than PET-CT [21]. Nevertheless, MRI can also produce false-positive and false-negative findings for a variety of reasons [22]. In the present case, contrary to the PET-CT findings, MRI found no evidence of a recurrence.

In addition, a meticulous physical examination was useful in determining that the area was not a recurrence but compensatory muscle hypertrophy of the tongue secondary to the treatment-related nerve paralysis. If the treatment and evaluation strategy had been based solely on imaging findings, unnecessary invasive tests, such as the biopsy of the site of FDG uptake on PET-CT, might have been performed. In general, imaging modalities are often relied upon for diagnosis, evaluation of treatment efficacy, and screening for recurrences of childhood malignancies. However, unlike in adult imaging tests, pediatric imaging tests involve a number of considerations. For example, children’s organs and tissues are more sensitive to radiation than those of adults and may have a higher risk of fatal, radiation-induced comorbidities [23]. Furthermore, infants in particular often require sedation to prevent artifacts associated with body movement, and clinicians should be aware of the risk of adverse events related to airway disturbances and hypoxia [24,25]. Therefore, imaging studies should be performed at the appropriate frequency using the optimal method for the purpose, and the clinician should confirm that the findings are consistent with the physical findings.

## 4. Conclusions

We discussed a case of false-positive FDG uptake on PET-CT caused by an unusual mechanism in a pediatric patient, which demonstrated the importance of taking detailed physical findings as well as imaging studies into account for diagnosis. Clinicians should determine carefully whether abnormal FDG uptake in a patient with a history of surgery indicates the recurrence of a malignancy.

## Data Availability

The data presented in this study are available in article.
